# Extra-Parotid Pleomorphic Adenoma and Low-Grade Salivary Malignancy in the Head and Neck Region

**DOI:** 10.7759/cureus.39463

**Published:** 2023-05-25

**Authors:** Diana Ann Jose, S.M. Azeem Mohiyuddin, Kouser Mohammadi, Prashanth Babu, Induvarsha G

**Affiliations:** 1 Otorhinolaryngology - Head and Neck Surgery, Sri Devaraj Urs Academy of Higher Education and Research, Kolar, IND

**Keywords:** recurrence, mucoepidermoid, adenoid cystic carcinoma, parapharyngeal space, extra-parotid salivary gland, pleomorphic adenoma

## Abstract

Background and objective

Pleomorphic adenoma is the most common major salivary gland neoplasm. Around 80% of them arise in the parotid glands, whereas 10% originate in the minor salivary glands. The pleomorphic adenoma of the extra-parotid site is defined by its location outside the primary salivary gland. The minor salivary gland adenomas occur at the hard and soft palate, lips, tongue, lacrimal glands, pharynx, larynx, paranasal sinus, and nasal cavity. Pleomorphic adenoma in parapharyngeal space may occur de novo or as an extension of the deep lobe of the parotid tumors. Our objective in this study was to assess the location and presentations of extra-parotid pleomorphic adenoma and frequency of low-grade salivary gland malignancy diagnosed as pleomorphic adenoma via fine-needle aspiration cytology (FNAC) in the head and neck region and the treatment outcomes after the resection of the tumors.

Materials and methods

This was a retrospective observational study. All patients with FNAC-diagnosed pleomorphic adenoma of extra-parotid locations of the head and neck region who underwent curative surgery in the Department of Otorhinolaryngology and Head and Neck Surgery at a rural tertiary care center between August 1997 and August 2022 were included in the study. Data on the symptoms, FNAC report, surgical techniques, pathological results, adjuvant therapy, and any recurrence were documented. Data were entered into a Microsoft Excel sheet and analyzed using IBM SPSS Statistics version 22 software (IBM Corp., Armonk, NY).

Results

Our study included 23 patients, of which 14 were females and nine were males. The various sites of involvement were as follows: parapharyngeal space (four), larynx (one), nasal septum (two), hard palate (five), soft palate (four), hard and soft palate (three), and submandibular salivary gland (four). Of note, 17.3% of the patients had local recurrence with an average time frame of three years post-surgery: 20% in patients with low-grade malignancy and 16.6% in patients with pleomorphic adenoma.

Conclusion

Extra-parotid pleomorphic adenomas are common and have a high malignant potential. The preferred choice of treatment for extra-parotid salivary tumors is complete resection with adequate clearance. Malignant pleomorphic adenomas may require staging neck dissection and adjuvant treatment for a better prognosis.

## Introduction

Salivary gland tumors are relatively uncommon, accounting for only 6% of head and neck tumors. They can be either benign or malignant. Pleomorphic adenoma is the most common benign tumor of the salivary glands; 65% of these cases affect the major salivary glands and 35% of cases involve the minor salivary glands [1]. The gold standard for the diagnosis of this condition is fine-needle aspiration cytology (FNAC). Low-grade malignancies might be misdiagnosed as pleomorphic adenoma. A few areas that are difficult to assess with FNAC may require incisional biopsy given the varied pathology of salivary gland tumors [2].

Of note, 0.5% of all head and neck neoplasms are found in the parapharyngeal space [3]. Pleomorphic adenoma in the parapharyngeal space might occur spontaneously or from the deep lobe of the parotid that extends via the stylo-mandibular tunnel. De novo pleomorphic adenoma in parapharyngeal space is uncommon and develops from salivary gland tissue abutting a lymph node [4]. Other sites include the palate, larynx, submandibular gland, nasal cavity, etc.

The key to effectively treating pleomorphic adenoma is extracapsular resection (clearance of the tumor with a sleeve of surrounding normal tissues) to avoid recurrence. Surgical approaches include complete resection via transpalatal, transmaxillary, mandibulotomy, transpterygoid, transoral or transnasal depending on the location, and some of these methods carry the risk of postoperative morbidity. Morbidity can be reduced by early diagnosis and a suitable surgical approach.

Predictors of malignant transformation include variation in consistency, rapid growth, pain and tenderness and regional lymphadenopathy, and facial nerve dysfunction [5]. The incidence of carcinoma ex-pleomorphic adenoma of a pre-existing benign pleomorphic adenoma is about 5-6%. The likelihood of a tumor becoming malignant increases with its longevity [6]. Adenoid cystic carcinoma is a malignant tumor more commonly involving minor salivary glands and has a protracted clinical course and poor prognosis. Lungs, bones, the liver, and the brain are the common sites of adenoid cystic carcinoma metastasis [7]. Although benign, regular long-term follow-up is necessary due to the possibility of local recurrence and malignant transformation. Extra-parotid low-grade malignancy might be diagnosed as pleomorphic adenoma by FNAC.

In this study, we aim to document the location and presentations of extra-parotid pleomorphic adenoma and frequency of low-grade salivary gland malignancy diagnosed as pleomorphic adenoma via FNAC in the head and neck region and the outcomes of treatment after the resection of the above-mentioned tumors.

## Materials and methods

In this retrospective observational study, all patients with FNAC-diagnosed pleomorphic adenoma of extra-parotid locations of the head and neck region and low-grade salivary malignancy who underwent surgery in the Department of Otorhinolaryngology and Head and Neck Surgery at a rural tertiary center were included. Informed consent to be a part of the study and Institutional Ethics Committee approval were obtained prior to the start of the study. Details regarding the demographic data, duration of swelling, location, size, depth, tenderness, consistency, fixity to surrounding structures, regional neck nodes, and cranial nerve involvement were documented. Patients underwent CT or contrast MRI to document the extent and relationship to adjoining vital structures. All patients underwent en-bloc resection of the tumors remaining extracapsular. The extent of the tumor, adequacy of surgical clearance, and malignant foci if any were documented by histopathological examination. Postoperative complications if any were documented. Patients were regularly followed up for three years to look for any recurrence. Data were entered into a Microsoft Excel sheet and analyzed using IBM SPSS Statistics version 22 software (IBM Corp., Armonk, NY). Categorical data were presented in the form of frequencies and via graphical representations.

## Results

**Figure 1:**
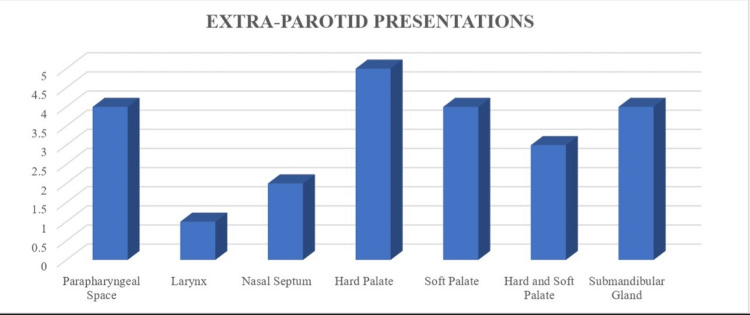
Graph showing various locations of extra-parotid pleomorphic adenoma

Our study included 23 patients with tumors in extra-parotid sites diagnosed as pleomorphic adenoma by FNAC. Among them, 14 were females and nine were males. Most of the patients were in the age group of 40-50 years. The various sites of involvement were as follows: parapharyngeal space (four), larynx (one), nasal septum (two), hard palate (five), soft palate (four), hard and soft palate (three), and submandibular salivary gland (four) (Figure 1).

**Figure 2:**
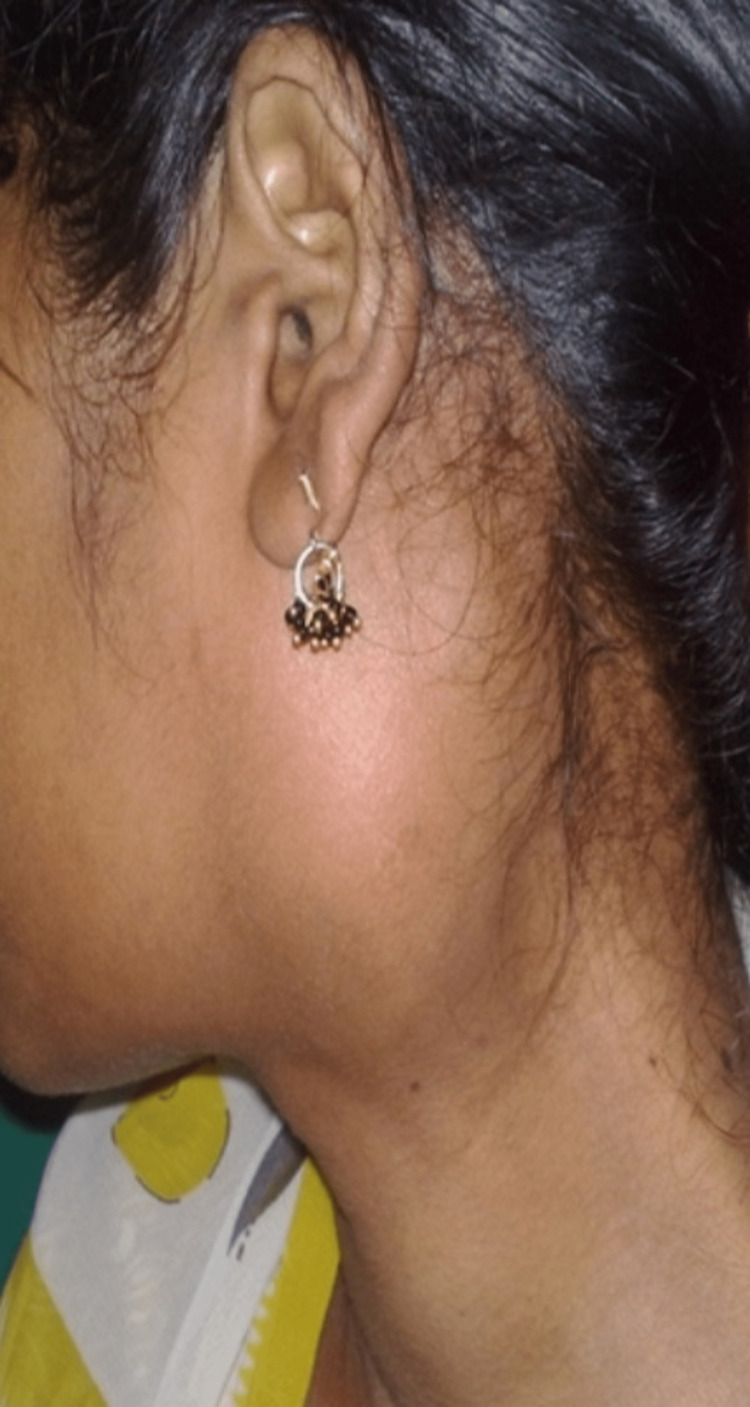
External presentation (infra-auricular extension) of parapharyngeal pleomorphic adenoma

Of the 23 patients, four presented with gradually progressive dysphagia and change in voice (hot-potato voice). Transoral examination showed a smooth bulge, firm in consistency over the soft palate extending and pushing the tonsil medially. Externally, a painless firm swelling was seen in the retromandibular extending to the infra-auricular region in three patients (Figure 2).

There was no extension to the nasopharynx and no cervical lymphadenopathy. The cranial nerve examination was normal. MRI revealed a mass in the parapharyngeal space with obliteration of fat planes and medialization of the lateral pharyngeal wall and reduced lumen in the oropharynx. FNAC from swelling showed evidence of pleomorphic adenoma. Complete resection of the mass was done by transcervical route with mandibulotomy approach as the tumor was extending high into masseteric space. An additional infra-temporal fossa clearance was done in two patients for an extension to this region. Postoperatively, two patients showed a temporary paresis of the marginal mandibular nerve (grade II palsy), which recovered within six months, and one patient with grade IV palsy, which improved after six months but residual grade II palsy persisted. Histopathology showed pleomorphic adenoma in all cases. One patient experienced recurrence after two years and underwent salvage resection.

**Figure 3:**
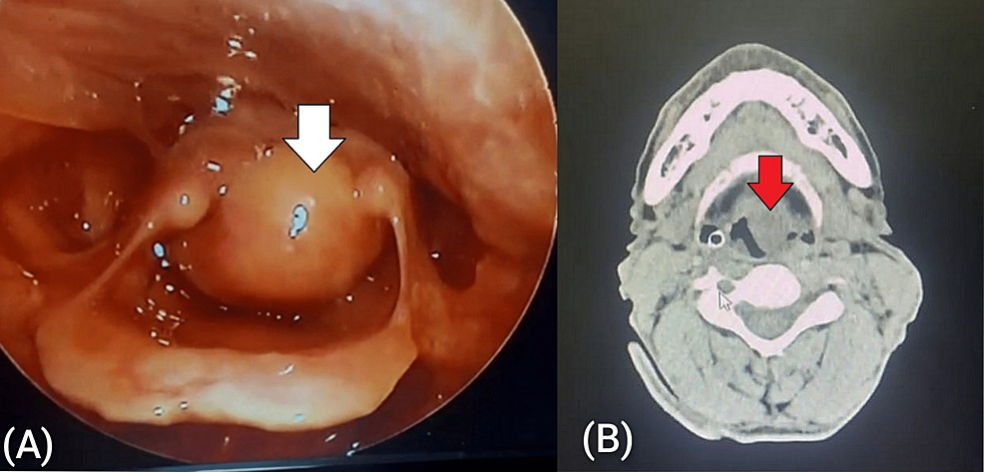
(A) Endoscopic image of laryngeal pleomorphic adenoma involving false cord; (B) CT image (axial section) of laryngeal pleomorphic adenoma CT: computed tomography

One patient presented with hoarseness of two-year duration and recent onset of stridor. On examination, there was a unilateral obstructive mass in the larynx (false cord and ventricle) (Figures 3A, 3B).

The patient underwent an emergency tracheostomy, and the mass was resected by microlaryngeal surgery. Histopathology showed pleomorphic adenoma. The patient was disease-free after three years of follow-up.

**Figure 4:**
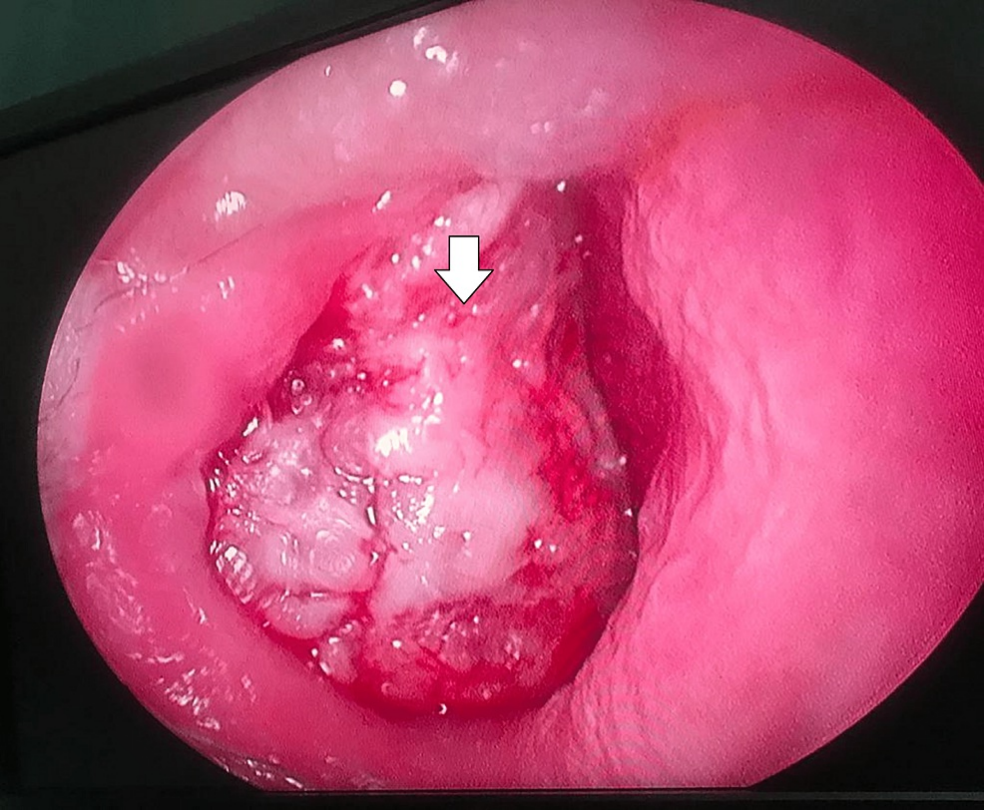
Endoscopic image of pleomorphic adenoma of the nasal septum

In the two patients who presented with nasal mass, rhinoscopy showed a fleshy mass arising from the nasal septum and filling the nasal cavity (Figure 4).

**Figure 5:**
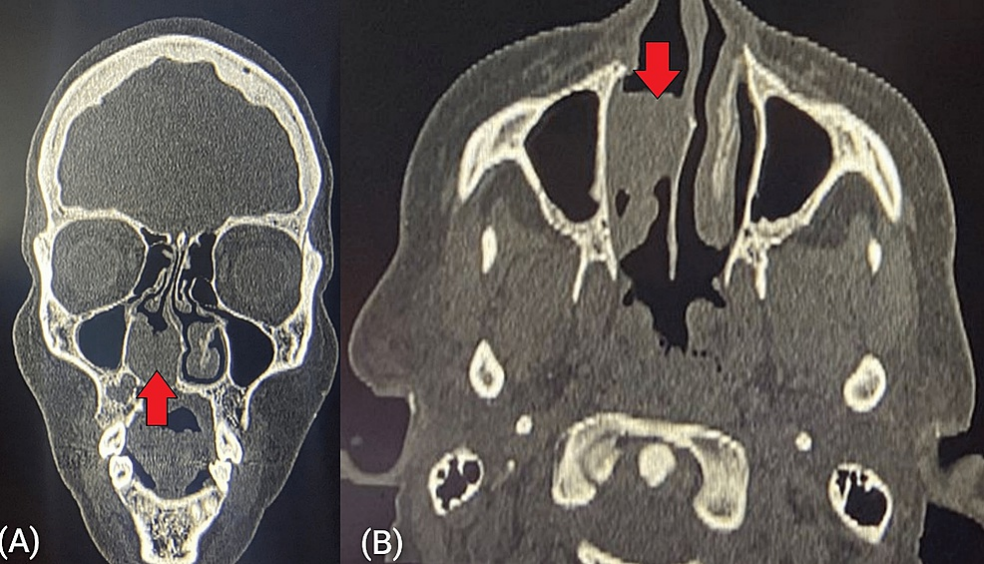
(A) CT image (coronal section) of pleomorphic adenoma of the nasal septum; (B) CT image (axial section) of pleomorphic adenoma of the nasal septum CT: computed tomography

Extension to the nasopharynx was seen in one case. CT confirmed the mass to be arising from the antero-basal part of the nasal septum in one case and involving the posterior part of the nasal septum in the second case (Figures 5A, 5B).

FNAC revealed a pleomorphic adenoma. Complete excision of the tumor was done transnasally in both cases with the partial removal of septal cartilage in one case. The septal defect was reconstructed using conchal cartilage. Histopathological examination of the resected specimen confirmed pleomorphic adenoma. On follow-up, the patient with antero-basal septal involvement had minimal nasal tip collapse; no septal perforation or recurrence was seen after five years of follow-up.

**Figure 6:**
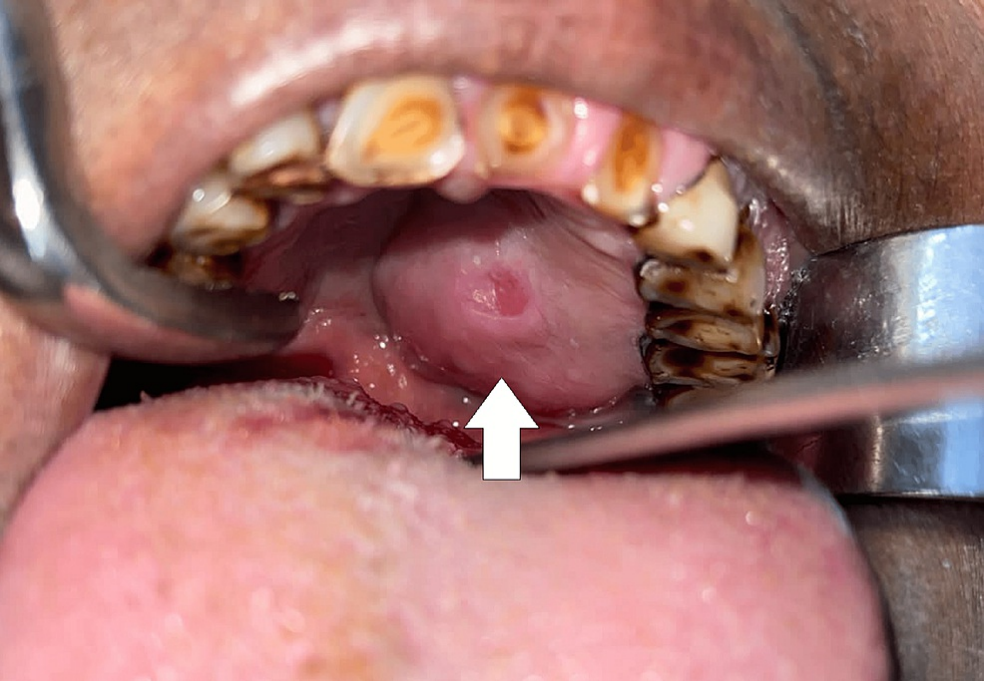
Mucoepidermoid carcinoma of the hard palate

**Figure 7:**
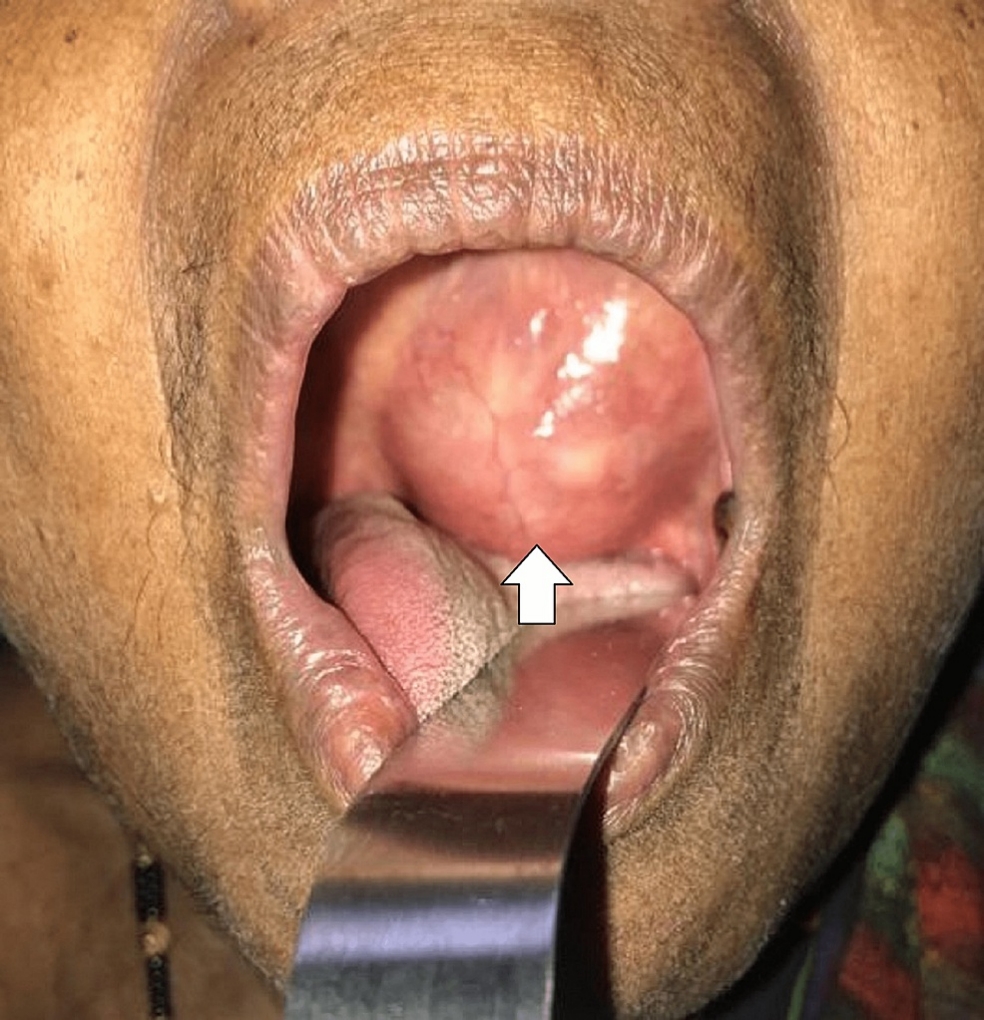
Pleomorphic adenoma of the soft palate

**Figure 8:**
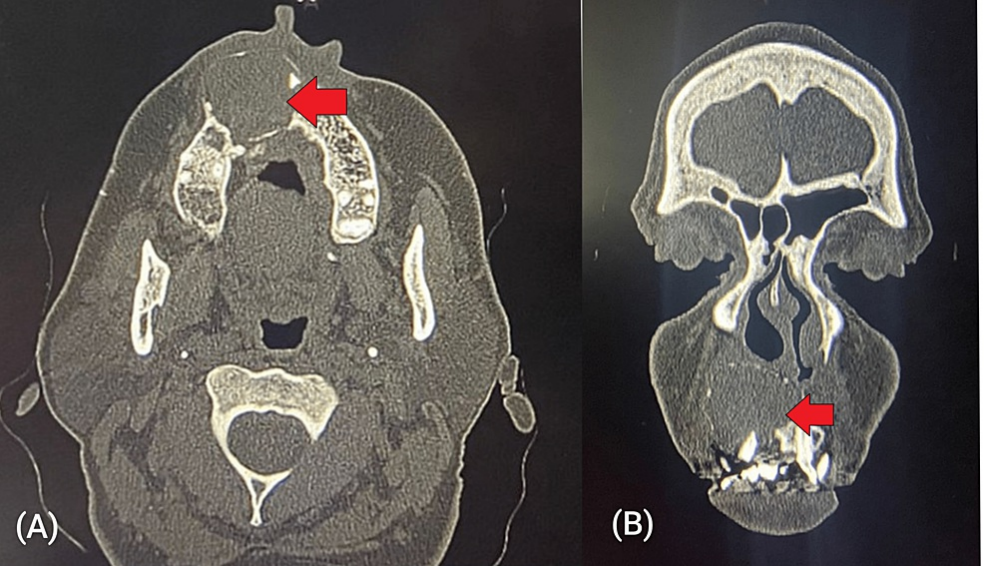
(A) CT image (axial section) of mucoepidermoid carcinoma of the hard palate; (B) CT image (coronal section) of mucoepidermoid carcinoma of the hard palate CT: computed tomography

Twelve patients presented with swelling in the palate (five in the hard palate, four in the soft palate, and three patients showing involvement in both hard and soft palates) (Figures 6, 7, 8A, 8B).

**Figure 9:**
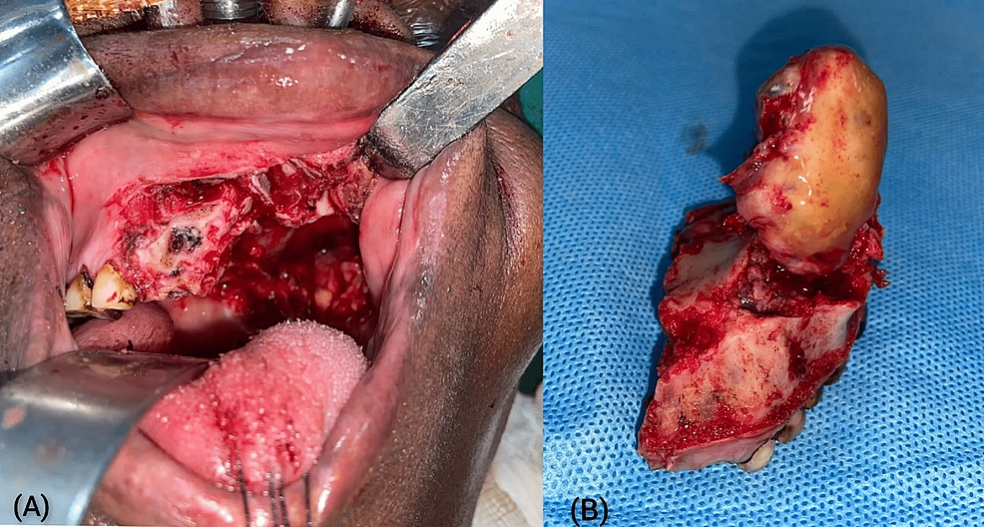
(A) Infrastructure maxillectomy procedure performed for a case of mucoepidermoid carcinoma of the hard palate; (B) Infrastructure maxillectomy specimen with tumor

All patients presented with firm, painless swelling. One patient showed extension into the superior part of the tonsillar fossa and lateral pharyngeal wall. FNAC showed pleomorphic adenoma. Complete excision was done by infrastructure maxillectomy (saving the orbital floor) with/without supraomohyoid neck dissection (SOHND) in five patients and the cavity was skin-grafted while the rest underwent transoral excisions (Figures 9A, 9B).

Postoperatively, an obturator plate was used for covering the defect. In one patient with soft palate involvement, extending to the anterior pillar and tonsillar fossa required coronoidectomy to facilitate a complete resection. The defect in the soft palate was repaired by either myomucosal advancement repair or uvuloplasty. Histopathology was suggestive of low-grade adenoid cystic carcinoma (tubular type) in one patient, low-grade mucoepidermoid carcinoma in two patients, and pleomorphic adenoma in all other patients. All three patients whose histopathology showed malignancy had tumors involving both the hard and soft palate. The patient with adenoid cystic carcinoma received adjuvant radiotherapy (60 Gy) but reported recurrence at the end of one year of follow-up and underwent total maxillectomy. Two patients who had pleomorphic adenoma involving the soft palate had local recurrence within three years of the follow-up period and underwent repeat resection.

**Figure 10:**
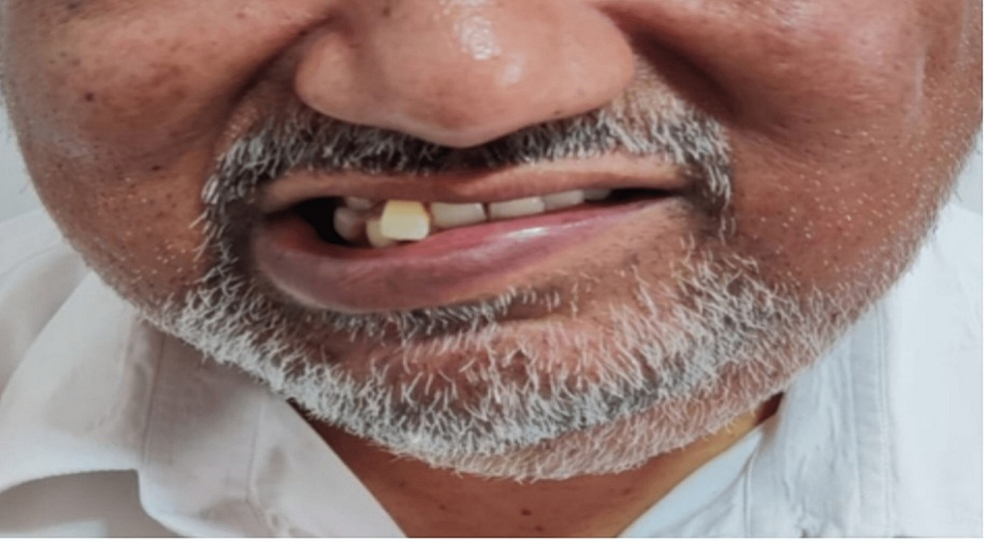
Patient with low-grade adenoid cystic carcinoma of the submandibular gland with marginal mandibular nerve palsy

Four patients presented with painless submandibular swelling. One of them had the marginal mandibular nerve and hypoglossal nerve palsy and extracapsular radical resection of the tumor with SOHND was performed (Figure 10).

Histopathology showed low-grade adenoid cystic carcinoma (tubular type) with perineural invasion and reactive lymph nodes. The patient received adjuvant radiotherapy; the patient had residual nerve palsy and was treated conservatively. Of the other three patients, one patient had low-grade mucoepidermoid carcinoma, and the remaining two patients were diagnosed with pleomorphic adenoma. All three patients underwent extracapsular resection. None of them showed recurrence on follow-up.

The various presentations of extra-parotid pleomorphic adenoma and low-grade salivary malignancy, preoperative FNAC results, treatment modality, and incidence of recurrence are presented in Table 1.

**Table 1:** Table showing presentation, treatment modality, and incidence of recurrence in patients with extra-parotid pleomorphic adenoma and low-grade salivary malignancy PPS: parapharyngeal space; ITF: infra-temporal fossa; SOHND: supraomohyoid neck dissection; ACC: adenoid cystic carcinoma; RT: radiotherapy; HPR: histopathological report; FNAC: fine-needle aspiration cytology

Sites	Preop FNAC	Surgery performed	HPR	Postop adjuvant therapy	Recurrence	Revision surgery
PPS (4 patients)	Pleomorphic adenoma	Complete resection by transcervical approach with mandibulotomy +/- ITF clearance	Pleomorphic adenoma	-	1 patient	Salvage resection
Larynx (1 patient)	Pleomorphic adenoma	Microlaryngeal surgery	Pleomorphic adenoma	-	-	-
Nasal septum (2 patients)	Pleomorphic adenoma	Complete transnasal excision with partial removal of septum	Pleomorphic adenoma	-	-	-
Hard palate (5 patients); soft palate (4 patients); hard and soft palate (3 patients)	Pleomorphic adenoma	5 patients: infrastructure maxillectomy +/- SOHND. 7 patients: transoral excision	1 patient: low-grade ACC (tubular type). 2 patients: low-grade mucoepidermoid carcinoma. 9 patients: pleomorphic adenoma	1 patient with ACC received adjuvant RT (60 Gy)	1 patient with ACC. 2 patients with pleomorphic adenoma	1 patient with ACC: total maxillectomy + RT. 2 patients with pleomorphic adenoma: repeat resection
Submandibular gland (4 patients)	Pleomorphic adenoma	Extracapsular radical resection in all 4 patients with SOHND in the patient with facial nerve palsy	1 patient: low-grade ACC (tubular type). 1 patient: low-grade mucoepidermoid carcinoma. 2 patients: pleomorphic adenoma	The patient with ACC received adjuvant RT (60 Gy)	-	-

## Discussion

Pleomorphic adenoma commonly occurs in the parotid gland; however, it can also occur in extra-parotid locations such as the palate, lips, buccal mucosa, tongue, tonsil, throat, retromolar region, parapharyngeal space, nasal cavity, and the larynx. According to the literature, females have a predisposition to benign salivary tumors [8]. Our study also had more female patients (60.8%) than males. As per Nagao et al., malignant salivary gland tumors are more common in males above 50 years of age [9]. A similar finding was noted in our study where 60% (three out of five) were male patients with malignant salivary gland tumors in the extra-parotid region.

Literature has shown that de novo parapharyngeal pleomorphic adenoma is uncommon [10]. Varghese et al. observed that misplaced or aberrant salivary gland tissue within a lymph node in the parapharyngeal region may be the cause [11]. The presentation includes an oropharyngeal mass, vocal changes, a trismus, an upper neck mass, cranial nerve deficits, and obstructive sleep apnea. In our patients, the most common finding was painless swelling in the parapharyngeal space, and only isolated cases had a change in voice and obstructive sleep apnea as presenting symptoms. Two main factors that adversely affect the complete excision of the tumor from the parapharyngeal space are difficult access and limited intraoperative exposure of the tumor for adequate extracapsular clearance, particularly in upper extensions like masticator space, and there is a need to minimize adverse functional or aesthetic outcomes. A variety of surgical approaches like transcervical-transparotid, transcervical, orbito-zygomatic, middle fossa, and mandibulotomy approaches, as well as combinations of these, can be used depending on the location and extent of the tumor [12].

De novo parapharyngeal space pleomorphic adenoma can also be excised by the submandibular transcervical approach with transoral excision via the tonsillar fossa approach [13]. Sergi et al. recommended a cervical transparotid approach and combined mandibular osteotomy for large parotid pleomorphic adenomas arising in the deep lobe parotid extending to the parapharyngeal space [5]. This approach also minimizes the chances of facial nerve palsy and provides better exposure to parapharyngeal space [4]. Stell et al., in their series of 58 patients with parapharyngeal tumors, advocated a cervical approach along with mandibulotomy [3]. The transcervical technique alone was recommended by Malone et al. and Hamza et al. [14,15]. In their study involving 172 patients, Hughes et al. observed that 94% of resections were performed by utilizing the transcervical and transparotid methods, with mandibular osteotomy being used in just 2% of cases [14]. All four patients in our study who had pleomorphic adenoma in parapharyngeal space were managed by cervical-transparotid approach with mandibulotomy. Postop histopathology revealed pleomorphic adenoma in all patients. One patient showed recurrence and was treated with salvage resection.

Glottic and supraglottic tumors of salivary origin presenting in the larynx can be resected by vertical partial laryngectomy or by transhyoid pharyngotomy, and posteriorly located tumors have been removed transorally by various authors [16,17]. In our study, such a tumor was removed by microlaryngeal surgery. The salivary tumors in the nasal cavity in our series were excised by various approaches, which included a septoplasty, mid-facial degloving approach, lateral rhinotomy, or endoscopic removal. Complete excision of the lesion with a circumferential cuff of normal septal mucosa is recommended. Local excision with/without septoplasty is preferred for intranasal/septal pleomorphic adenoma [1,2]. This is also recommended for infiltrating adenoid cystic carcinoma [6]. In our study, both the salivary gland tumors were found to be pleomorphic adenomas and one of them was extending into the nasopharynx and was excised transnasally along with a partial resection of the septal cartilage.

The frequency of adenoid cystic carcinoma in the minor salivary glands is high. Two of our patients with submandibular pleomorphic adenoma on FNAC underwent complete resection of the submandibular gland with SOHND as a staging procedure in view of marginal mandibular and hypoglossal nerve palsy and enlarged intraoperative nodes respectively. The histopathological report confirmed low-grade adenoid cystic carcinoma in the former while mucoepidermoid carcinoma in the latter. In view of perineural invasion and reactive lymph nodes in histopathology, adjuvant radiotherapy was given to the patient with adenoid cystic carcinoma.

As per the literature, the prognosis of adenoid cystic carcinoma of the submandibular gland is good with early diagnosis, extensive surgical intervention, and postoperative radiotherapy. Adverse prognosis is associated with presentations with an advanced tumor, positive surgical margins, and perineural invasion [18]. Three of our patients with pleomorphic adenoma of the palate on FNAC had CT features showing cystic components with significant lymph nodes, and the intraop frozen section showed low-grade malignancy. Elective SOHND was planned along with infrastructure maxillectomy. The postoperative histopathological report confirmed low-grade adenoid cystic carcinoma in one patient, low-grade mucoepidermoid in two patients, and the rest had pleomorphic adenoma. Adenoid cystic carcinoma of the hard palate requires infrastructure maxillectomy, preferably with adjuvant radiation. A case series has shown the presence of lung metastasis, which highlights the importance of screening for distant metastasis and the need for adjuvant chemotherapy [19].

In spite of administering adjuvant radiotherapy (60 Gy) for a patient with adenoid cystic carcinoma, at the end of two years of follow-up, the patient developed local recurrence and was managed by revision total maxillectomy. Despite complete locoregional control, some studies have shown local recurrence as well as delayed distant (lung and cutaneous) metastasis in spite of aggressive treatment and adjuvant treatment [7]. Two patients who had pleomorphic adenoma involving the soft palate also presented with local recurrence at the end of three years and were successfully managed by revision surgery.

Although the preoperative diagnostic FNAC showed pleomorphic adenoma, postoperative histopathology revealed low-grade adenoid cystic carcinoma and low-grade mucoepidermoid carcinoma. This disparity is not common in the literature. However, low-grade malignancy can be mistaken for pleomorphic adenoma. Therefore, in case of suspicion, it is better to have guided FNAC preferably confirmed by two different pathologists.

Local recurrence, malignant transformation, and metastasis are the primary concerns in extra-parotid salivary tumors. Recurrence rates vary from 0 to 8% [1]. In our study, 17.3% (4/23) of patients had local recurrence within an average duration of three years post-surgery: 20% (one out of five) in patients with malignancy and 16.6% (3/18) in patients with pleomorphic adenoma. They were managed by salvage resection and adenoid cystic carcinoma was radiated. No patient presented with metastasis. The probable reasons for recurrence could be the tumor's close proximity to neurovascular structures, and extension to difficult-to-access areas (upper part of masseteric space), which could have led to residual pseudopodal extensions being left behind. Aggressive salivary gland tumors have microscopic extensions beyond visible margins notorious for perineural invasion. The chance of malignant transformation is 6% and is predicted to increase by 1.5% every year after five years [1]. Tyrosine kinase inhibitors have been shown to be effective in treating inoperable recurrences of adenoid cystic carcinoma, with response rates ranging from 0 to 11% [20].

Limitations

Our study was a single-center study with a small study population. Determining the exact incidence of extra-parotid pleomorphic adenoma and low-grade salivary malignancy in the Indian population will require studies with a larger study population with the involvement of multiple centers.

## Conclusions

Extra-parotid pleomorphic adenomas are frequently encountered and have a high chance of malignancy. Complete resection is the first line of treatment for extra-parotid salivary tumors and adequate exposure during surgery is mandatory. Adenoid cystic carcinoma requires adjuvant treatment and mucoepidermoid carcinoma may require neck dissection. Low-grade malignancy and benign tumors carry a good prognosis following complete extracapsular resection. Tumors located in the high upper part of the masseteric space and infratemporal fossa may require mandibulotomy for better exposure.
